# IFNL3 mRNA structure is remodeled by a functional non-coding polymorphism associated with hepatitis C virus clearance

**DOI:** 10.1038/srep16037

**Published:** 2015-11-04

**Authors:** Yi-Fan Lu, David M. Mauger, David B. Goldstein, Thomas J. Urban, Kevin M. Weeks, Shelton S. Bradrick

**Affiliations:** 1 Department of Molecular Genetics and Microbiology, Duke University Medical Center, Durham NC, 27710, USA; 2 Department of Chemistry, University of North Carolina, Chapel Hill, NC 27599-3290, USA; 3 Institute for Genomic Medicine, Columbia University, New York, NY 10032, USA; 4 Center for Pharmacogenomics and Individualized Therapy, Eshelman School of Pharmacy, University of North Carolina, Chapel Hill, NC 27599-7361, USA; 5 Department of Biochemistry and Molecular Biology, University of Texas Medical Branch, Galveston, TX 77555, USA

## Abstract

Polymorphisms near the *interferon lambda 3* (IFNL3) gene strongly predict clearance of hepatitis C virus (HCV) infection. We analyzed a variant (rs4803217 G/T) located within the IFNL3 mRNA 3′ untranslated region (UTR); the G allele (protective allele) is associated with elevated therapeutic HCV clearance. We show that the IFNL3 3′ UTR represses mRNA translation and the rs4803217 allele modulates the extent of translational regulation. We analyzed the structures of IFNL3 variant mRNAs at nucleotide resolution by SHAPE-MaP. The rs4803217 G allele mRNA forms well-defined 3′ UTR structure while the T allele mRNA is more dynamic. The observed differences between alleles are among the largest possible RNA structural alterations that can be induced by a single nucleotide change and transform the UTR from a single well-defined conformation to one with multiple dynamic interconverting structures. These data illustrate that non-coding genetic variants can have significant functional effects by impacting RNA structure.

Human genetic variation has a large influence on an individual’s susceptibility to infectious diseases. Studies on the hepatitis C virus (HCV), a major cause of liver disease^1^, have revealed a striking example of how human genetics affects the outcome of infection. Genome-wide association studies performed on diverse patient populations have identified polymorphisms near the interferon-λ3 (IFNL3; formerly IL28B) gene that predict the efficacy of interferon-based therapy for chronic infection. These polymorphisms are also strongly predictive of spontaneous HCV clearance during the acute phase of infection^2^,^3^,^4^,^5^.

IFNL3 is a secreted cytokine that binds a specific cell surface receptor complex expressed on epithelial cells, leading to JAK-STAT signal transduction and expression of interferon-stimulated genes^6^,^7^,^8^. IFNLs have been implicated in control of viral infections of epithelial-derived tissues, such as gut, lung, and liver^9^,^10^,^11^,^12^. IFNLs inhibit replication of multiple viruses *in vitro*^13^,^14^,^15^ and IFNL3 genotype has been linked to liver interferon-stimulated gene mRNA expression in chronic HCV patients and primary human hepatocytes^16^,^17^,^18^.

The mechanisms by which IFNL3 genetic variants function to control HCV infection are not clear. Steady-state levels of IFNL3 mRNA in liver tissue and peripheral blood mononuclear cells may be linked to HCV clearance because the protective alleles (rs12979860 C and rs8099917 T) are correlated with a modest (<2-fold) increase in IFNL3 mRNA levels^3^,^4^,^19^,^20^. However, other studies on different patient populations and cell types failed to detect correlations between differences in IFNL3 mRNA expression and genotype in liver tissue^16^,^17^ or plasmacytoid dendritic cells^21^. Thus, the propensity for HCV clearance appears to be related to differences beyond IFNL3 mRNA levels.

Although primary hepatocyte cultures have been shown to express IFNL3 upon HCV infection^9^,^22^, evidence is emerging to support a role for dendritic cell subtypes in the production of IFNL3^23^,^24^,^25^. In addition, levels of IFNL3 protein were found to be strongly correlated with IFNL3 genotype in serum samples from HCV-infected patients^26^,^27^. One study also suggested that serum IFNL3 levels are a stronger predictor of viral clearance than IFNL3 genotype^28^. These observations, taken together, suggest that IFNL3 abundance may be regulated by differential efficiency of mRNA translation as a function of IFNL3 genotype.

Here we report the genetic association and functional consequences of a SNP (rs4803217) located in the 3′ untranslated region (UTR) of IFNL3 mRNA. This SNP was previously reported to regulate IFNL3 expression by changing seed base pairing with HCV-induced microRNAs^29^. We genotyped a large number of patients with chronic hepatitis C from the IDEAL Cohort^30^ to evaluate associations of this variant with treatment phenotypes. Molecular analyses suggested that rs4803217 is functional in regulation of mRNA translation efficiency. RNA structure probing by SHAPE provided a physical explanation for these functional differences and revealed that IFNL3 mRNA structure is markedly altered by this common SNP. Together, these findings support a model in which IFNL3 mRNA translation efficiency, governed by allele-specific mRNA structures, modulates clearance of HCV infection.

## Results

### The rs12979860 discovery SNP and rs4803217 are highly correlated and exhibit indistinguishable associations with clinical phenotypes

**Figure 1:**
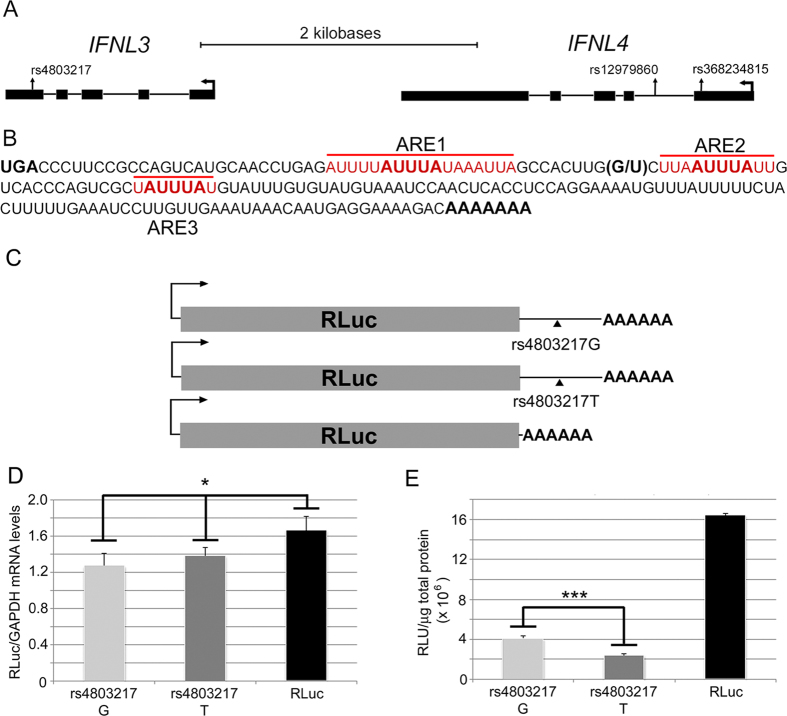
Variant IFNL3 3′ UTRs differentially inhibit reporter gene expression. (**A**) IFNL genes and genetic variants. (**B**) The IFNL3 mRNA 3′ UTR sequence. The three AREs (1–3) are indicated in red and the rs4803217 SNP is bracketed. (**C**) IFNL3 reporter constructs. Transcription of each reporter mRNA is directed by the constitutive CMV promoter. (**D**) Relative levels of each RLuc reporter mRNA. RNA levels were normalized to GAPDH; asterisk indicates that differences are significant at the p < 0.05 level. (**E**) RLuc protein levels. Protein levels were measured by luciferase assay and normalized to total protein. Triple asterisks indicate that differences are significant at the p < 0.001 level. All data are shown as mean values ± s.d.

The most strongly associated single nucleotide polymorphisms (SNPs) found in genome-wide association studies (GWAS) were rs12979860 in European-Americans and African-Americans, and rs8099917 in East Asians. These SNPs likely co-segregate with polymorphisms that directly affect biological functions related to IFNL expression or activity. To identify candidate functional variants in the IFNL3 gene region, we searched for variants in high linkage disequilibrium with rs12979860 (hereafter referred to as the ‘discovery SNP’) by analysis of available human genome sequence data (1000genomes.org). We identified a SNP (rs4803217) in the 3′ UTR of IFNL3 that is highly correlated (best tagging SNP according to 1000 Genomes Project) to the discovery SNP^31^,^32^,^33^,^34^ (Fig. 1A). The rs4803217 G allele is correlated with the protective C allele of the discovery SNP. This variant was an intriguing candidate as the source of viral infection-related phenotypes because 3′ UTRs often contain *cis*-acting RNA elements that control mRNA translation and decay. Rs4803217 is also flanked by AU-rich elements (AREs; Fig. 1B), sequence motifs that have been linked to post-transcriptional gene regulation^35^. Therefore, we conducted a large-scale genetic association study examining rs4803217 and HCV clearance in a cohort of chronically-infected patients.

We genotyped and analyzed these variants in the IDEAL Cohort^30^, a large sample of chronic HCV patients treated with pegylated IFN-α and ribavirin combination therapy (n = 792 European-American; n = 169 African-American). The sample sizes of both populations are sufficient to achieve greater than 0.99 in statistical power (1.00 in European-American and 0.992 in African-American under the significance level of 0.01). Linkage disequilibrium analysis in HCV patients revealed that rs4803217 is strongly associated with rs12979860 in European-American patients (r^2^ > 0.97; Table 1), with somewhat lower linkage disequilibrium in African-Americans (r^2^ = 0.913). We performed association tests of rs4803217 with clinical phenotypes using logistic regression. The association of rs4803217 with sustained virological response (defined as absence of detectable HCV RNA in serum at least 24 weeks after discontinuation of treatment) was extremely significant (p = 2.48 ×10^−25^) in the European-American population (Table 1). The discovery SNP has been previously associated with viral burden such that patients with the protective allele (C) exhibit a slightly higher baseline viral load^2^. We found that rs4803217 exhibited comparable association with pre-treatment baseline viral load by linear regression in both European- and African-Americans compared with the discovery SNP (Table 2).

Next, we performed a multiple logistic regression analysis to examine whether rs4803217 showed independent association with patient phenotypes in excess of that attributed to the discovery SNP. This analysis revealed that rs4803217 did not show significant association with SVR when we adjusted for rs12979860 genotype, in either European- or African-Americans (Table 1). Thus, although the IDEAL cohort is one of the largest and most data-rich clinical trial cohorts available for genetic studies of patient response to IFN-based therapy, the extremely high linkage disequilibrium between variants in this region precluded independent statistical association of rs4803217 with SVR in this sample size.

Recently, a functional dinucleotide SNP rs368234815 that can influence the expression of IFNL4 protein was discovered upstream of the IFNL3 locus and was implicated in the clearance of HCV^36^. In addition, the putatively favorable rs4803217 G allele was proposed to have an unexpected negative effect on the decrease of HCV RNA level after treatment in the presence of the unfavorable rs368234815 ΔG allele^37^, suggesting that rs4803217 may modify the effect of the rs368234815 variant associated with IFNL4. Here, we also performed the same multiple logistic regression or multiple linear regression approaches to assess whether rs4803217 or rs368234815 showed stronger association with patient phenotypes. We found that, despite the large sample size of the IDEAL cohort, this analysis did not reveal significant independent association of either SNP after correcting for the effect of each other (Table 1 and Table 2), although we note an interesting opposite trend of odds ratios (OR) for SVR after mutual adjustment (0.78 and 2.64 for rs4803217 G and rs368234815 TT, respectively) in African American patients (Table 1).

Overall, rs4803217 demonstrated similar levels of association with patient phenotypes as did the discovery SNP or a nearby functional SNP rs368234815, suggesting that rs4803217 is an interesting candidate variant and may be responsible for clinically important phenotypic differences.

### rs4803217 impacts IFNL3 mRNA translation efficiency

Although rs4803217 did not independently predict SVR in patients, based on association evidence it is a good candidate to investigate. Furthermore, its presence in the IFNL3 mRNA 3′ UTR prompted us to test functionality. The IFNL3 3′ UTR is 176 nucleotides (nt) in length, is composed of 65% A and U nucleotides, and harbors several functional AREs^29^, including two (ARE1 and ARE2) flanking rs4803217 (Fig. 1B). Comparison of UTRs from primate IFNL3 and IFNL2 genes indicates that the region around rs4803217 is highly conserved and that the T variant is the ancestral allele (Supplementary Figure S1).

We first evaluated the effects of the variant IFNL3 3′ UTRs on steady-state mRNA and protein levels using a reporter assay. We took advantage of a stable HeLa cell line that harbors a genomic flippase recognition target site^38^ to create isogenic cell lines with distinct *Renilla* luciferase (RLuc) reporter constructs controlled by a CMV promoter that is transcriptionally regulated by tetracycline repressor protein. This system allows characterization of gene expression in cells containing a single copy of the reporter gene. Three cell lines were established containing either RLuc alone or RLuc fused to each of the two IFNL3 3′ UTR variants (Fig. 1C). The presence of either IFNL3 3′ UTR caused modest but statistically significant (p = 0.028 for G allele, p = 0.046 for T allele, unpaired t-test) reductions in the levels of IFNL3 chimeric mRNAs compared to RLuc alone (Fig. 1D). Interestingly, levels of the RLuc protein were reduced by a much larger amount, 4- and 7-fold for the rs4803217 G and T reporters, respectively, compared to the reporter lacking an IFNL3 3’ UTR (Fig. 1E). The differential effects on mRNA versus protein levels strongly suggest that the IFNL3 3′ UTR regulates gene expression by repressing the efficiency of mRNA translation rather than mRNA abundance in HeLa cells. Importantly, the 3′ UTR SNP affected the magnitude of this repression: the rs4803217 T allele produced approximately 50% less RLuc than did the corresponding G allele mRNA (Fig. 1E; p = 0.0005, unpaired t-test). Induction of reporter mRNA expression levels by tetracycline treatment reduced RLuc differences between the cell lines, suggesting that reporter mRNA over-expression led to escape from endogenous regulatory factor(s) present in HeLa cells (Supplementary Figure S2A,B). We also tested the IFNL3 reporter constructs in an immortalized human hepatocyte cell line (PH5CH8), a human hepatoma cell line (LH86), and HeLa cells by transient plasmid transfection (Supplementary Figure S2C). In each cell line, the patterns of reporter expression mirrored that in the stable HeLa cell lines (Fig. 1E).

**Figure 2:**
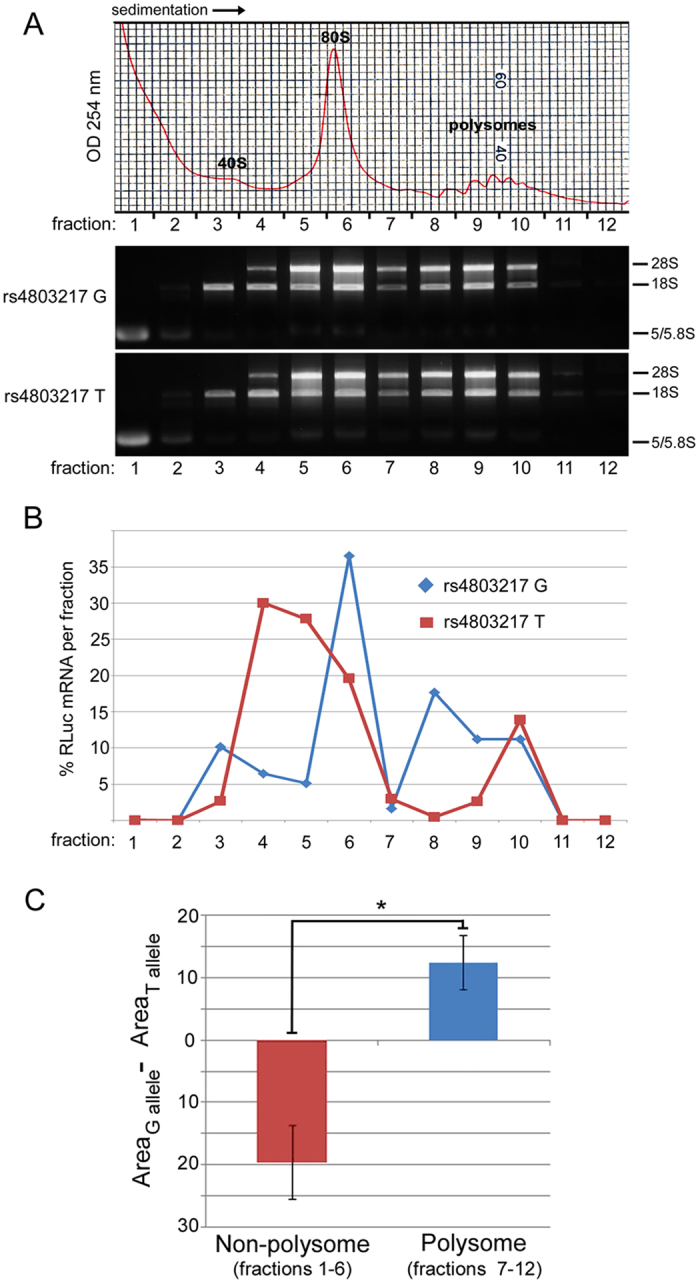
Polysome profiles of IFNL3 reporter mRNAs. (**A**) Top: Representative ribosome profiles. Locations of the 40S subunit, 80S monosome, and polysome peaks are indicated. Profiles were obtained using a sucrose density gradient, monitored by absorbance trace (254 nm). Bottom: Analysis of extracted total RNA samples in each gradient fraction for each IFNL3 reporter cell line. Ribosomal RNA species are indicated at right. (**B**) Relative levels of RLuc reporter mRNA normalized to GAPDH mRNA for each IFNL3 cell line as a function of ribosome gradient fraction. The relative levels (%) of each mRNA in each gradient fraction are indicated. (**C**) Areas underneath the lines in Figures 2B and S3 were quantified for both polysome fractions (fractions 7–12) and non-polysome fractions (fractions 1–6). The plot shows the differences in area between the G and T alleles (G minus T). Unpaired t-test (two-tailed) was used to calculate the P value from the three independent experiments (p = 0.01). Error bar represents the SEM.

We next directly tested the hypothesis that rs4803217 regulated efficiency of mRNA translation. We used polysome profiling to examine whether the variant IFNL3 reporter mRNAs were differentially associated with translating ribosomes in stable HeLa cell lines. The extent of association with multiple ribosomes is an indication of mRNA translation efficiency. IFNL3 variant reporter cell lines, grown to equal densities, were harvested, lysed and subjected to sucrose density gradient centrifugation to separate non-translating and ribosome-associated mRNA. Gradients were subsequently fractionated and RLuc reporter and GAPDH mRNA levels were measured by RT-qPCR. Ribosomal RNA sedimentation patterns from the IFNL3 two reporter cell lines were nearly identical (Fig. 2A), indicating that bulk mRNA translation rates did not differ between cell lines. In contrast, the rs4803217 G and T IFNL3 reporter mRNAs showed distinct profiles across the gradient. Across three biological replicates, the G allele mRNA was more highly represented in denser gradient fractions, reflecting incorporation into polysomes, than the T allele mRNA (Fig. 2B,C and Supplementary Figure S3). We quantified the areas underneath the lines for both G and T alleles and found that the allelic difference in area between polysome fractions (fractions 7–12) and non-polysome (fractions 1–6) fractions were significantly different (p = 0.01) (Fig. 2C). This result indicates that the G allele mRNA was more abundant in polysome fractions compared to the T allele mRNA. Correspondingly, levels of non-translating mRNA (sedimenting between the 40S ribosomal subunit and the 80S monosome) were higher for the rs4803217 T mRNA. We did note that a significant amount (15%) of the T-allele reporter mRNA was found in dense gradient fractions (10–12), suggesting that a portion of the T allele reporter mRNA escapes repression and is efficiently translated. Combined with the discrepant luciferase protein and reporter mRNA measurements shown in Fig. 1, these data strongly suggest that the rs4803217 SNP regulates efficiency of IFNL3 reporter mRNA translation in HeLa cells.

### rs4803217 alters structural conformation of the IFNL3 3′ UTR

There is extensive literature linking 3′ UTRs with translational control [see reference^39^ for a recent review]. A growing body of evidence indicates that 3′ UTR structure regulates mRNA translation and decay by modulating interactions with *trans*-acting factors^40^. Moreover, genetic variants that alter mRNA structure and function, termed RiboSNitches, have been described^41^. We directly examined whether the rs4803217 SNP physically modulates the structure of endogenously expressed IFNL3 mRNA using selective 2′-hydroxyl acylation analyzed by primer extension (SHAPE)^42^. We examined the structure of IFNL3 mRNA under non-denaturing conditions in the context of total purified cellular RNA. SHAPE analysis was performed on deproteinized RNA as this approach allows for direct assessment of mRNA structure independently of *trans*-acting proteins or RNAs. The structure of the IFNL3 mRNA was probed using the fast-reacting SHAPE reagent 1-methyl-7-nitroisatoic anhydride (1M7) and products were analyzed by massively parallel sequencing using the SHAPE-MaP strategy^43^ (Supplementary Figure S4). We used human A549 lung adenocarcinoma cells for these experiments because they express IFNL3 mRNA upon transfection with poly(I:C) (Supplementary Figure S5A). Importantly, A549 cells are heterozygous at rs4803217 and levels of mRNA from each allele were directly correlated with the gene dosage (Supplementary Figure S5B). Using the MaP approach, sequencing reads derived from IFNL3 were sorted by SNP identity at rs4803217; this made it possible to examine the structures of both alleles under identical conditions in the same experiment (Supplementary Figure S4). SHAPE data were collected on a 266 nt long region of the IFNL3 mRNA containing the last 125 nt of the coding sequence (including the stop codon) and the 3′ UTR, excluding the terminal 23 nt and the poly(A) tail.

**Figure 3:**
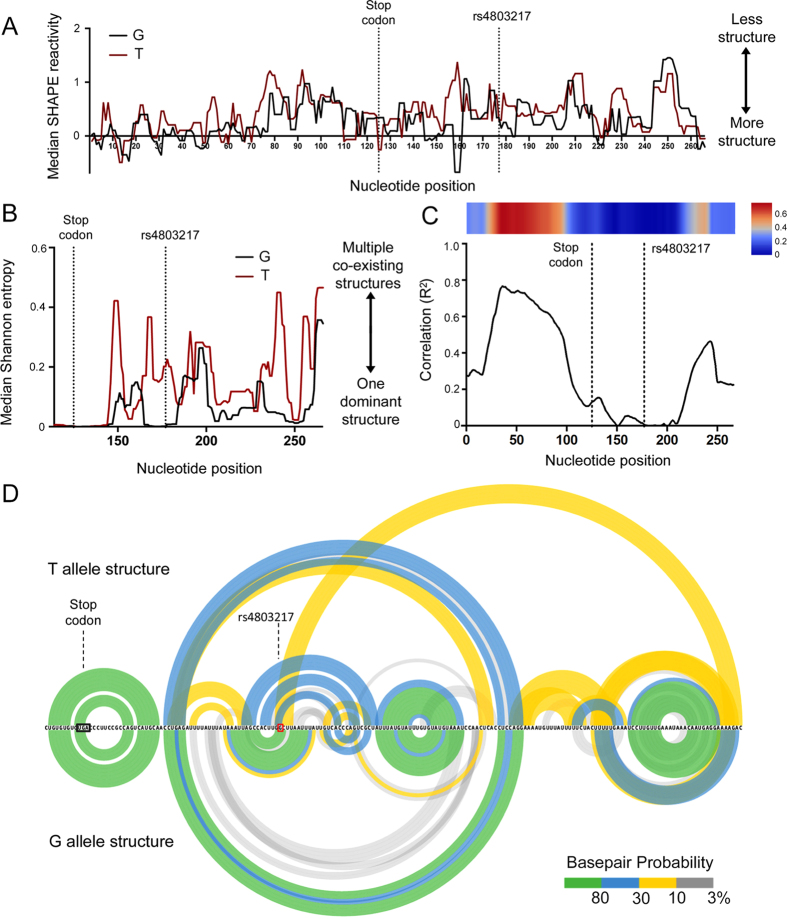
SNP-induced structural changes in the IFNL3 3′ UTR. (**A**) SHAPE reactivity profiles. Low median SHAPE reactivities correspond to highly structured regions in the RNA. Reactivities are shown as the median over 5-nt windows. “Nucleotide position” values indicate nt locations in the context of the total SHAPE data (**B**) Shannon entropies (medians over 5-nt windows). Peaks indicate regions with high Shannon entropies and that likely adopt multiple conformations. (**C**) Correlation of median SHAPE reactivities between the G and T alleles. R^2^ was calculated over 100-nt windows. (**D**) SHAPE-directed RNA secondary structure models for the 3′ UTR. Base pairs are shown as arcs. Arcs are colored by pairing probability.

SHAPE-MaP reactivities report a model-free measurement of the degree of RNA structure^42^,^43^. The rs4803217 G allele had lower SHAPE reactivity, and thus more stable structure, near the region close to rs4803217 than did the T allele (Fig. 3A). We next calculated Shannon entropy values for each rs4803217 allele. Shannon entropies are derived from a SHAPE-directed partition function and report a measure of whether an RNA region is likely to form a single well-defined structure^43^,^44^,^45^. The median Shannon entropies across the IFNL3′** UTR were much lower for the G allele, especially near the rs4803217 site, than for the T variant (Fig. 3B and Supplementary Figure S6). We next performed a correlation analysis of SHAPE reactivities between the G and T alleles by calculating R^2^ across the mRNA regions to address the overall structural similarity between the two alleles (Fig. 3C). The SHAPE reactivities in the open reading frame (ORF) are highly correlated between alleles, and moderately correlated at the very end of 3′ UTR. In contrast, a very low or zero correlation was observed in the region of rs4803217. This indicates that a drastic local change in structure is induced by the SNP. In sum, the G allele 3′ UTR adopts an overall well-defined, highly structured conformation; whereas, nucleotide-resolution SHAPE probing indicates that the T allele adopts multiple conformations. In addition, structural differences between the two 3′ UTR alleles are centered on the SNP region.

SHAPE data can be used to develop accurate models for large, complexly structured RNAs with well-defined structures^46^,^47^. SHAPE-directed RNA structure models for each variant over the 3′ portion of the open reading frame and 3′ UTR are summarized in arc plots that display predicted short- and long-range base pairing (Fig. 3D). Given its low Shannon entropy, the structure of the G allele could be modeled with a high degree of confidence, as evidenced by the preponderance of highly probable helices (Fig. 3D, bottom). In contrast, the 3′ UTR for the T allele is predicted to adopt multiple conformations, comprised of helices with base pairs of lower individual probability (Fig. 3D, top). The combined SHAPE reactivity and entropy differences indicate that the T-allele mRNA has a more variable structure than the G-allele mRNA.

**Figure 4:**
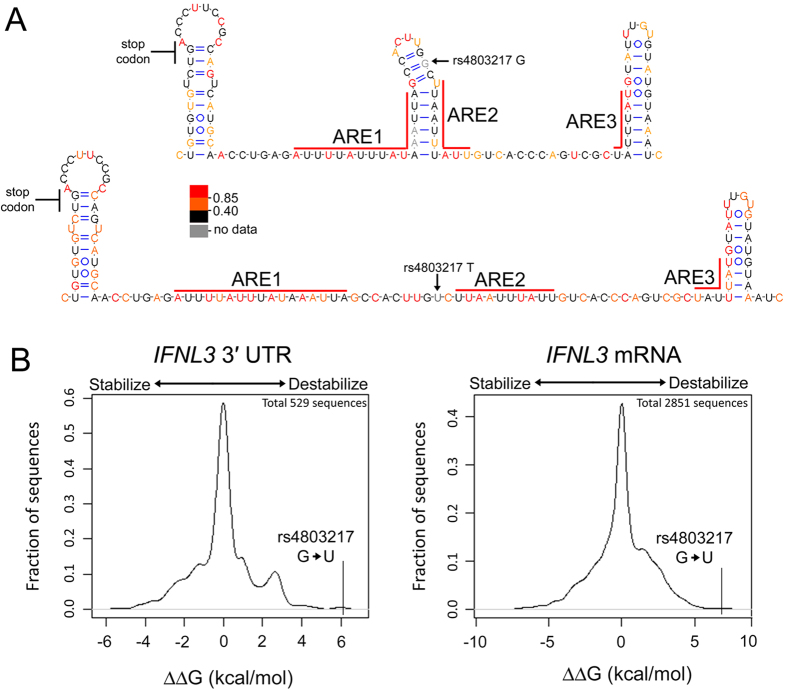
Secondary structure models for the IFNL3 3′ UTR. (**A**) SHAPE-directed RNA secondary structure models of the IFNL3 3′ UTR G (top) and T (bottom) alleles. rs4803217 site is shown for each structure. Nucleotides are colored by SHAPE reactivity. Highly probable (>80%) helices for each allele are shown. Locations of AU-rich elements are indicated on each allele structure. (**B**) Calculated free energy change increments (ΔΔG) for all possible nucleotides changes in the IFNL3 3′ UTR (left) or complete intact mRNA (right). Batch calculation was performed by mfold 3.0.

The SHAPE-directed RNA secondary structure model indicated that the rs4803217 G nucleotide forms a canonical base pair within a stable stem-loop (Fig. 4A and Supplementary Figure S7) that contains portions of ARE1 and ARE2. In contrast, the U variant is not predicted to stably adopt this stem-loop structure (Fig. 4A). We calculated the free energy change of 3′ UTR RNA folding^48^ for each allele and found that the rs4803217 G to U change alters relative free energy by approximately 7 kcal/mol. This difference in relative free energy is close to the largest possible increment achievable by altering a single base pair^49^. We calculated the expected free energy changes for all possible nucleotide changes in the context of the IFNL3 3′ UTR (529 sequence variants) and in the context of the intact mRNA (2851 sequence variants). The increase in folding free energy due to the change from G to U at rs4803217 ranked the highest among all possible substitutions in 3′ UTR, and the third among all possible substitutions in the intact mRNA, indicating that this position has a strong influence on RNA folding (Fig. 4B). Direct SHAPE experimental interrogation and free energy increment calculations thus suggest that the rs4803217 SNP induces close to the largest possible change in RNA structure.

**Figure 5:**
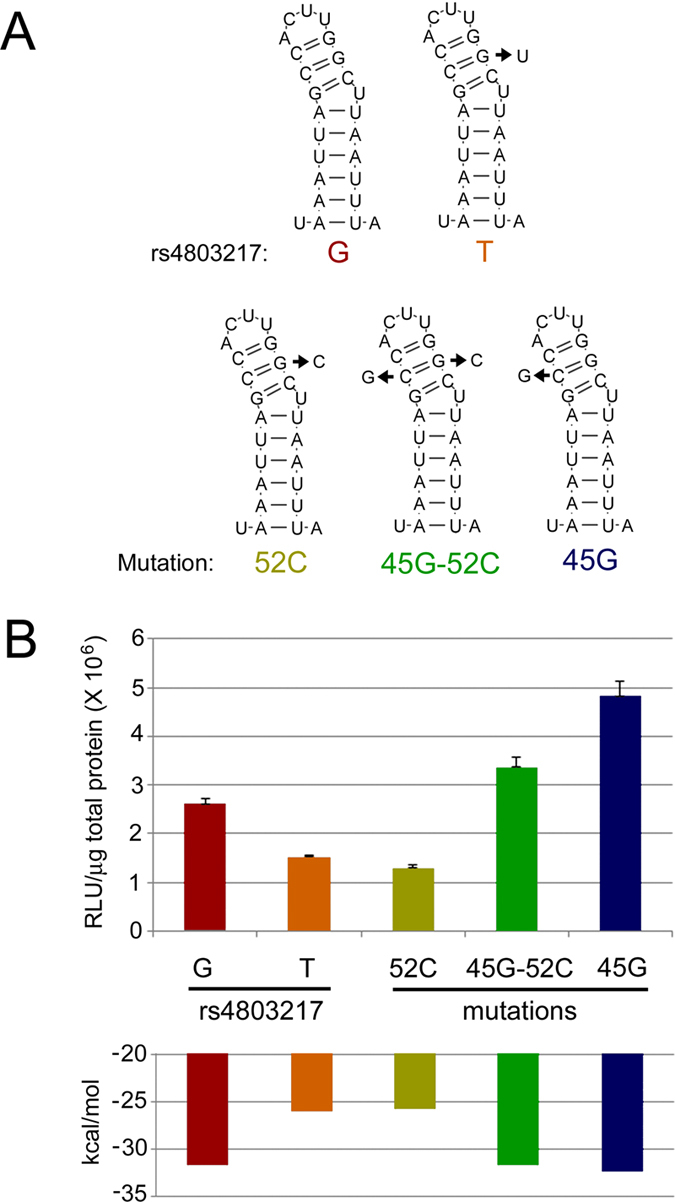
Site-directed mutagenesis of the IFNL3 3′ UTR. (**A**) The rs4803217 G and T allele reporter constructs were compared to three mutants in stable HeLa cell lines. (**B**) Normalized relative light units (RLU) for each reporter construct is shown above. Data are shown as mean values ± s.d. and folding free energies for the full 3′ UTR sequence are shown.

We performed mutagenesis to further test whether functional differences for rs4803217 reflect changes in RNA structure. Three mutant versions of the IFNL3 reporter construct were established that contained mutations at the rs4803217 position (nt 52 of the 3′ UTR) and/or at the nt position predicted to base-pair with rs4803217 (nt 45 of the 3′ UTR) (Fig. 5A). Changing rs4803217 from G to C resulted in reduced expression, similar to the rs4803217 T reporter construct (Fig. 5B). Restoring the predicted base pair in this mutant by introducing a G at nt 45 increased expression, consistent with RNA structure opposing repression. Interestingly, introduction of G at nt 45 (C45G) in the context rs4803217 G, which is predicted to interrupt base-pairing, had increased expression which was opposite of the effect on expression initially expected. However, this mutant resulted in a free energy of RNA folding similar to the rs4803217 G 3′ UTR (Fig. 5B) by inducing a register shift in base pairing and ultimately preserved the stem loop associated with escape from repression^48^ (Supplementary Figure S8). For each of the constructs analyzed, the expression levels were inversely correlated with folding free energy. Together, the mutagenesis data further reinforce the model that the T-allele has a more variable structure than the G-allele mRNA and that stable structure is associated with enhanced HCV clearance.

## Discussion

Multiple genome-wide association studies have identified polymorphisms near the IFNL3 gene that predict efficacy of therapy and spontaneous HCV clearance^2^,^3^,^4^,^5^. Although rs4803217 is not independently associated with patient phenotypes in the cohort we analyzed, this SNP occurs in the 3′ UTR of IFNL3 and showed clear functional effects on reporter gene expression, suggesting a role for this variant in control of HCV infection.

Rs4803217 influences regulation of mRNA translation efficiency by the IFNL3 3′ UTR in HeLa cells, perhaps through altering functionality of AREs. Although cytokine 3′ UTR AREs are commonly associated with enhanced mRNA decay, they have also been implicated as translational regulators^50^,^51^,^52^,^53^ and mRNA decay and translation are closely associated cytoplasmic processes^54^,^55^. Due to gene regulation at the level of mRNA translation, the modest differences in IFNL3 mRNA levels, as a function of genotype, reported in some studies may underestimate actual differences in IFNL3 protein levels, as we observed here for the IFNL3 rs4803217 SNP (Fig. 1). This is consistent with a recent report of ~ 7-fold differences in IFNL2/3 serum levels between HCV patients who are homozygous at the discovery SNP^26^. Consistent with this work, a recent independent study identified a distinction between CC versus non-CC genotype (discovery SNP) for IFNL3 protein plasma levels^27^. However, we acknowledge that our results do not formally establish a causal relationship between rs4803217 and IFNL3 levels in patients.

To examine and define the physical basis for the mechanism of IFNL3 translational regulation, we used the SHAPE-MaP strategy, which made it possible to resolve structural conformations and differences for the rs4803217 G and U variant mRNAs in a single heterozygous cell line, in a single experiment. We discovered that the nucleotide at rs4803217 influenced the global structure of IFNL3 mRNA. The G allele was much more highly structured and the free energy difference between the G- and U-containing mRNAs was predicted to be close to the upper limit in structural stabilities achievable by a single-nucleotide sequence change.

Our work strongly suggests that rs4803217 is a RiboSNitch^41^ that alters gene expression by inducing changes in RNA structure. Recent genome-wide analyses suggest that RiboSNitches are widespread in the human transcriptome^56^. The experimental and computational strategies developed here can be applied to other candidate functional variants to advance our understanding of how post-transcriptional gene regulation is affected by non-coding genetic polymorphisms. These approaches should be broadly useful for identifying human genome sequence variants in non-coding regions that exert functional effects by altering RNA structure.

The observed change in protein expression is likely the result of differential binding of *trans*-acting factors, induced by the differential folding of the 3′ UTR variants. The SHAPE-derived secondary structures show that rs4803217 T reduces structure of a region overlapping ARE1 and 2 (Fig. 4A), suggesting that unidentified trans-acting ARE-binding proteins may access these elements more efficiently in the T allele mRNA. Recently, McFarland *et al.* reported that the IFNL3 mRNA is regulated by AU-rich elements and also targeted by muscle-specific micro(mi)RNAs (miR-208b and miR-499a-5p) whose expression is triggered by HCV replication. These authors found that rs4803217 was functional using IFNL3 reporter mRNAs. However, the SNP did not confer regulation when ARE1 or ARE2 were mutated, in agreement with our observation that rs4803217 alters structural conformation of regions encompassing ARE1 and ARE2. With respect to the mechanism involving action of miRNAs, the rs4803217 T to G allele would disrupt miRNA-mRNA seed base-pairing^29^. Thus, the dramatic effects of rs4803217 on IFNL3 mRNA structure that we report here may regulate interactions with ARE-binding proteins and miRNAs. However, we note that, when the SNP position was mutated to a C, we observed a level of reporter expression similar to that of the T-allele (Fig. 5B). The rs4803217 C mutation abolishes seed-region base-pairing to the proposed regulatory miRNAs, similar to the G-allele. This suggests that the regulation we characterized in HeLa cells, which may lack expression of the implicated miRNAs, is mediated primarily by effects of RNA structure on interaction with *trans*-acting factor(s) other than miR-208b or miR-499a-5P. Further study is needed to define the complement of relevant *trans*-acting factors that may differentially regulate variant IFNL3 mRNA transcripts through repression of translation and/or induction of mRNA decay.

Recently, RNA sequencing analysis of primary human hepatocytes (PHH) revealed transcriptional activity upstream of the IFNL3 gene that is coincidentally induced by treatment with poly I:C^36^. Moreover, a dinucleotide variant (rs368234815 TT/Δ G) in this region is associated with HCV clearance and may alter translation of a specific mRNA isoform encoding the novel IFNL4^36^. IFNL4 is antiviral against HCV *in vitro* yet is poorly secreted and does not appear to be strongly expressed in PHHs^15^,^57^. In the rs368234815 ΔG genotype, which is predicted to express IFNL4 and is in high linkage disequilibrium (LD) with the non-protective (T) rs12979860 allele, IFNL4 production may exert effects that would be unexpectedly deleterious to HCV clearance^36^. Rs368234815 was recently reported to be more strongly associated with some patient phenotypes than rs4803217 in different patient cohorts, and thus may be dominant in determining HCV clearance in patients^37^. Further study is needed to understand the potentially maladaptive effects of IFNL4 expression and dissect possible interactions between rs4803217 and ss469415590 variants in control of HCV infection.

In summary, we demonstrate a general strategy involving genetics, molecular biology, and RNA chemistry for understanding GWAS associations with non-coding genetic variants that may alter RNA structure and post-transcriptional gene regulation. Nucleotide-resolution SHAPE probing revealed that the 3′ UTR of the rs4803217 G allele IFNL3 mRNA, which is associated with HCV clearance, forms a well-defined structure whereas that of the T allele mRNA is dynamic. The large alteration induced by a single nucleotide change illustrates the extent to which non-coding genetic variants can have significant functional effects by impacting RNA structure.

## Methods

### Genotyping and statistical analysis

DNA from the IDEAL cohort was used for genotyping. The genotyping of rs4803217 was performed by quantitative PCR (Bioline) using, forward primer 5′-ACCTG AGATT TTATT TATAA ATTAG CCACT TG(G/T)-3′, and reverse primer 5′-CTTTT CCTCA TTGTT TATTT CAACA AGGAT TTC-3′. Data were clustered by Ct value for genotype calling. Rs368234815 was genotyped by the TaqMan genotyping assay (Life Technologies) according to the protocol recommended by the manufacturer, forward primer 5′-TGGGT CCTGT GCACG GTGAT-3′, reverse primer 5′-TCCCT CAGCG CCTTG GCA-3′, and probe 5′-CGCAG (AA/C)GGCC CCCCG G-3′^15^. Statistical analysis of phenotypic data was performed in STATA (StataCorp) and R (www.r-project.org). Sustained virological response (SVR) was used as the binary dependent variable, and genetic polymorphisms were considered independent variables in the logistic regression model (the assumption of logistic regression requires the dependent variable to be binary). Both discovery SNP and rs4803217 were used as independent variables in multiple logistic regression to distinguish its independent association after accounting for the effect of each other. Viral loads (IU/ml) were transformed by Box-Cox method and considered as the dependent variable in linear regression to meet the multivariate normality assumption. Sample size calculation was performed by the pwr package in R and the significance level was set at p = 0.01 under the multiple regression setting. All the p values reported in this paper are based on the two-sided statistical tests.

### Cell culture, cloning and RT-qPCR

PH5CH8^58^, LH86^59^, A549 and stable HeLa-FRT cells expressing TetR were cultured in high glucose DMEM supplemented with 10% heat-inactivated FBS and non-essential amino acids. Stable HeLa-FRT cells (a gift from Elena Dobrikova, Duke University) harboring RLuc reporter trans-genes were established as described^38^ by co-transfection of a flp recombinase plasmid (pOG44, Life Technologies) with RLUC reporter plasmids cloned into pcDNA5/FRT/TO (Life Technologies, see below). Stable cell lines were selected with blasticidin (2.5 μg/ml) and hygromycin (200 μg/ml). Analysis of RLuc levels (*Renilla* Luciferase Assay System, Promega) in HeLa cell lines was conducted in the absence or presence of tetracycline (1 μg/ml) induction. Stable reporter cell lines were independently derived and analyzed three times. Reporter constructs were generated by PCR amplification of the RLuc open reading frame using 5′–GAGGT ACCAT GACTT CGAAA GTTTA TGATC C-3′ and 5′–GAGAT ATCTT ATTGT TCATT TTTGA GAACT CGC-3′ and ligation into pcDNA5/FRT/TO (Life Technologies) using *Kpn*I and *EcoR*V. The resulting clone was used for insertion of IFNL3 3′ UTR sequences PCR amplified from A549 genomic DNA using *EcoR*V and *Not*I (primers: 5′-GAGAT ATCCC CTTCC GCCAG TCATG CAACC TGAG-3′ and 5′-GAGCG GCCGC CGCAC ACACA GTCCC ACGTC ATGGG T-3′). Mutant constructs were established by cloning custom synthesized DNA fragments (Integrated DNA Technologies). All clones were verified by Sanger sequencing. For RT-qPCR, total RNA samples were obtained by RNeasy column purification using on-column DNase treatment (Qiagen). RNA samples were converted to cDNA (Life Technologies) and quantified by qPCR (Power SYBR Master Mix, Life Technologies; Applied Biosystems Step One Plus instrument). A minus RT control was included to control for contaminating genomic DNA. Primer sequences for qPCR were: GAPDH_F: 5′-AGCCA CATCG CTCAG ACAC-3′, GAPDH_R: 5′-GCCCA ATACG ACCAA ATCC-3′, RLUC_F: 5′-CAGTG GTGGG CCAGA TGTAA ACAA-3′, RLUC_R: 5′-TAAGA AGAGG CCGCG TTACC ATGT-3′.

### Cell transfections

Transient transfections of PH5CH8, LH86, or HeLa cells were performed using the pcDNA5/FRT/TO reporter plasmids described above. Cells were plated onto 24-well plates at a density of 7.5 × 10^5^ cells/well 24 hours prior to co-transfection with 50ng (LH86, HeLa) or 100ng (PH5CH8) of RLuc reporter plasmids and 20 ng of pGL3 firefly luciferase plasmid (Promega) using 0.5 μl Lipofectamine 2000 (Life Technologies). Cells were lysed 24 hours after transfection and luciferase measurements were made using by dual luciferase assay (Promega). All reporter plasmids were analyzed in triplicate and P values were calculated by unpaired t-test.

A549 transfections for RT-PCR analysis of IFNL3 expression were performed with 7.5 × 10^5^ cells plated 24 hours prior to transfection into 24-well plates. Cells were transfected with 800 ng pcDNA5/FRT/TO plasmid, poly I:C (Sigma), or purified HCV JFH1 strain^60^ RNA produced by *in vitro* transcription with T7 RNA polymerase (Ambion). Four hours after transfection, total RNA samples were generated using Trizol reagent (Life Technologies) and used for standard RT-PCR detection of IFNL3 mRNA using primers designed to amplify the open reading frame.

### Polysome profiling

HeLa cell lines were cultured on 10 cm dishes and processed essentially as described^61^. Briefly, cells were washed, scraped and pelleted in ice-cold PBS containing 200 μM cycloheximide. Cell pellets were resuspended in 0.4 ml lysis buffer [400 mM KOAc, 25 mM K-HEPES (pH 7.5), 15 mM Mg(OAc)_2_, 1 mM DTT, 200 μM cycloheximide] and incubated on ice for 30 minutes before centrifugation for 10 minutes at 12,000 × g to remove insoluble material. Supernatants were layered onto 10 ml 15–50% sucrose density gradients containing 400 mM KOAc, 25 mM K-HEPES (pH 7.5) and 15 mM Mg(OAc)_2_ and then centrifuged for 3 hours at 35,000 rpm using an SW41 rotor. Gradients were fractionated (Isco) and 12 fractions were collected for RNA extraction using Trizol LS (Life Technologies). Polysome analysis was conducted with single replicate per cell line in three biological replicates conducted on different days.

### RNA SHAPE analysis

Adherent A549 cells were plated in a 6-well culture dish and grown overnight to 90% confluency. IFNL3 mRNA was induced by transfecting cells (using Lipofectamine 2000) with 4 μg of poly I:C (Sigma) and incubated for 4 hours before washing with PBS (pH 7.2).

For SHAPE modification of total deproteinized mRNA, RNA was purified using a modified Trizol (Invitrogen) extraction protocol. The cells were lysed with 1.0 mL of Trizol for 5 min at room temperature. The cell lysate was placed in a microfuge tube and 0.2 mL of chloroform was added followed 15 seconds of shaking vigorously by hand. The sample was incubated for 2 min at room temperature, and the sample was spun in the centrifuge at 12,000 × g for 15 min. The deproteinized total RNA in the aqueous layer was removed and immediately equilibrated into native folding buffer [50 mM HEPES (pH 8.0), 5 mM MgCl2, 200 mM potassium acetate] using a pre-equilibrated G-25 spin column (GE Health sciences). Deproteinized RNAs were incubated at 37 ^o^C for 15 minutes in native folding buffer in order to allow folding state to come to equilibrium and then treated with 0.1 volume of DMSO, or 100 mM 1-methyl-7-nitroisatoic anhydride (1M7) in DMSO at 37 ^o^C for 5 min. EDTA was added to 10 mM final concentration; reactions were chilled on ice; and the RNA was precipitated with isopropanol. For the parallel denaturing modification control, deproteinized total RNA was equilibrated into 1X denaturing buffer [50 mM HEPES (pH 8), 4 mM EDTA, 50% formamide] using a pre-equilibrated G-25 spin column; heated to 95 ^o^C for 1 min; modified with 0.1 volume of 100 mM 1M7 in DMSO; chilled on ice for 2 min; and recovered by precipitation with isopropanol.

### SHAPE-MaP library construction

DNA libraries for massively parallel sequencing were prepared as described^62^. Briefly, targeted SHAPE-MaP reverse transcription reactions (20 μL) contained 2 μM of IFNL3 RT primer (5′-GTCTT TTCCT CATTG TTTAT TTC-3′) in 1× SHAPE-MaP buffer [50 mM Tris-HCl (pH 8), 75 mM KCl, 6 mM MnCl_2_] with 1 μL reverse transcriptase (Superscript II, Invitrogen). Reactions were incubated at 42 °C for 3 hours, and cDNA products were purified (G-25 microspin column; GE Lifesciences). Purified cDNAs were amplified for 20 cycles using Q5 DNA Polymerase (NEB) with Illumina adapted IFNL3 targeted primers (Forward 5′-GACTG GAGTT CAGAC GTGTG CTCTT CCGAT CNNNN NCCTC CACCA TTGGC TGC-3′, Reverse: 5′-CCCTA CACGA CGCTC TTCCG ATCTN NNNNN NNCTC ATTGT TTATT TCAAC AAGGA TTTC-3′). The forward primer was designed with a 3′ mismatch to the IFNL2 mRNA to limit amplification of this similar transcript. PCR primers were designed to amplify sequences spanning different exons, so analyzed PCR product could only be generated from a spliced mRNA and not contaminating genomic DNA. PCR products were purified using a PureLink PCR purification kit (Invitrogen), and libraries were amplified by a second PCR reaction using Q5 DNA Polymerase (NEB) and Illumina TRUseq PCR primers. PCR libraries were quantified by fluorescence (Qubit fluorometer; Life Technologies), analyzed with a Bioanalyzer DNA kit (Agilent), and sequenced on a MiSeq instrument (Illumina).

### SHAPE-MaP data analysis

SHAPE-MaP reactivities for each nucleotide within the IFNL3 3′ UTR were generated from the raw FASTQ files. Raw SHAPE reactivity data are shown in Table S1. A custom analysis pipeline identified all of the sequencing reads from IFNL3 rs4803217 G, IFNL3 rs4803217 T, and IFNL2 mRNAs. IFNL2 reads were determined by analyzing six nucleotide positions that vary between IFNL2 and IFNL3. Reads with three or more nucleotide matches to IFNL2 (1.2% of total reads) were removed prior to RNA structure analysis. Reads from the two IFNL3 alleles were sorted into separate FASTQ files and then analyzed using ShapeMapper^62^. Shannon entropies and minimum-free energy genome secondary structure models were generated from SHAPE reactivities using a custom RNA folding pipeline^62^. Shannon entropy values were calculated to quantify the well-determinedness of structural states for a given RNA region. The pipeline interfaces with RNAstructure v5.5 in order to computationally model RNA secondary structure. The RNA secondary structure models were diagramed using VARNA^63^.

## Additional Information

**How to cite this article**: Lu, Y.-F. *et al.* IFNL3 mRNA structure is remodeled by a functional non-coding polymorphism associated with hepatitis C virus clearance. *Sci. Rep.* **5**, 16037; doi: 10.1038/srep16037 (2015).

## Supplementary Material

Supplementary Information

Supplementary Information

## Figures and Tables

**Table 1:** Association of rs4803217 with SVR in the IDEAL cohort.

	r^2^*	OR**	P	P (after correcting for rs12979860)	r^2^*	OR**	P	P (after correcting for rs12979860)
**Discovery SNP in GWAS**								
rs12979860-C	-	3.92	4.18 × 10^−25^	-	-	2.10	0.006	-
**Candidate functional variant**								
rs4803217-G	0.977	3.93	2.48 × 10^−25^	0.219	0.913	1.77	0.030	0.100
rs368234815-TT	0.977	3.96	1.81 × 10^−25^	0.118	0.708	2.13	0.004	0.328
	**European-American N** **=** **792**				**African-American N** **=** **169**			
**Comparison between functional SNPs**	r^2^ (between functional SNPs)	OR**	P		r^2^ (between functional SNPs)	OR**	P	
	OR**	P						
rs4803217-G (correcting for rs368234815-TT)	0.965	1.69	0.393		0.701	0.78	0.629	
rs368234815-TT (correcting for rs4803217-G)		2.37	0.158			2.64	0.065	

Logistic regression or multiple logistic regression were performed by additive genetic model.

*r^2^ represents pairwise LD with GWAS discovery SNP (rs12979860).

**OR = odds ratio.

**Table 2:** Association of rs4803217 with the pre-treatment viral load.

	coefficient	P	(after correcting for rs12979860)	coefficient	P	P (after correcting for rs12979860)
**Discovery SNP in GWAS**						
rs12979860-C	12.89	7.81 × 10^−7^	-	20.68	5.41 × 10^−6^	-
**Candidate functional variant**						
rs4803217-G	12.61	1.35 × 10^−6^	0.793	21.25	2.25 × 10^−6^	0.196
rs368234815-TT	13.03	5.77 × 10^−7^	0.412	22.20	7.57 × 10^−7^	0.037
	**European-American N** **=** **792**			**African-American N** **=** **169**		
**Comparison between functional SNPs**	coefficient	P		coefficient	P	
rs4803217-G (correcting for rs368234815-TT)	-5.79	0.677		9.04	0.253	
rs368234815-TT (correcting for rs4803217-G)	18.71	0.178		14.61	0.066	

Association tests were performed by linear regression or multiple linear regression (additive genetic model).
