# Supplementary material for: IFNL3 mRNA structure is remodeled by a functional non-coding polymorphism associated with hepatitis C virus clearance
Source: Sci Rep. 2015 Nov 4;5:16037. doi: 10.1038/srep16037 (PMC4631997; doi:10.1038/srep16037)

Supplementary Information

IFNL3 mRNA structure is remodeled by a functional non-coding polymorphism associated with hepatitis C virus clearance

Yi-Fan Lu, David M. Mauger, David B. Goldstein, Thomas J. Urban, Kevin M. Weeks and Shelton S. Bradrick

Supplementary Figure Legends

Supplementary Figure 1. Alignment of primate IFNL2 and IFNL3 sequences. Location of the rs4803217 SNP is indicated. AREs are underlined in the consensus sequence.

Supplementary Figure 2. Analysis of IFNL3 reporter constructs on HeLa, PH5CH8, and LH86 cells. (A) Stable HeLa cell lines described in Figure 1 were induced to express the indicated reporter mRNAs by treatment with tetracycline and cell lysates were analyzed for luciferase activity at 2 and 3 hours after tetracycline treatment. Data from untreated cells is shown as the "0" hour time point. Data are shown as mean values ± s.d. (B) Same as in panel (A) showing only the untreated cells. (C) PH5CH8, LH86, and HeLa cells were transiently transfected with the indicated reporter plasmids as described in the Materials and Methods. For each cell line, the RLuc value was set to equal 1. P values were calculated by unpaired t-test (*** p = 0.018, ** p = 0.021, * p = 0.030).

Supplementary Figure 3. Polysome profiles of *IFNL3* reporter mRNAs. Two additional independent replicates of the polysome profile experiment from Figure 2 are shown.

Supplementary Figure 4. SHAPE-MaP deconvolution of multiple alleles. Total cellular RNA was isolated from poly(I:C)-transfected A549 cells and treated with 1M7 reagent after RNA purification. Modified or DMSO-treated (control) RNA samples were subjected to IFNL3-targeted SHAPE-MaP. Resulting sequencing reads were sorted for nucleotide identity at the rs4803217 locus and used to generate structural profiles for each individual allele.

Supplementary Figure 5. A549 cells inducibly express IFNL3 mRNA and are heterozygous at rs4803217. (A) RT-PCR analysis of IFNL3 mRNA expression in A549 cells. Cells were either untreated (M), or transfected with vector plasmid (DNA), poly(I:C) (pI:C), or

synthetic HCV genomic RNA from the JFH1 strain (HCV). Total RNA was harvested 4 hours after transfection and analyzed by RT-PCR and agarose gel electrophoresis. (B) Analysis of A549 DNA and RNA amounts for the IFNL3 3' UTR rs4803217 SNP. DNA and RNA samples were analyzed by qPCR and RT-qPCR, respectively, using an assay that discriminates rs4803217 G versus T/U alleles. A549 cells are triploid at IFNL3 with two alleles for rs4803217 T and one for G. mRNA levels reflect the gene dosage.

Supplementary Figure 6. Analysis of median Shannon entropy as a function of window size: (A) 3 nt, (B) 11 nt, or (C) 21 nt. For reference, Figure 3 uses a window size of 5 nts.

Supplementary Figure 7. SHAPE-directed minimum free energy secondary structure for the IFNL3 “G” allele 3′ UTR. The structure includes 11 nt of coding sequence including the stop codon (in red). Location of rs4803217 is indicated by an arrow.

Supplementary Figure 8. Predicted structure of the C45G mutant stem loop. The C45G mutation is indicated in red and location of rs4803217 is shown. Note the predicted re-folding of the stem loop due in the C45G mutant.

Figure S1

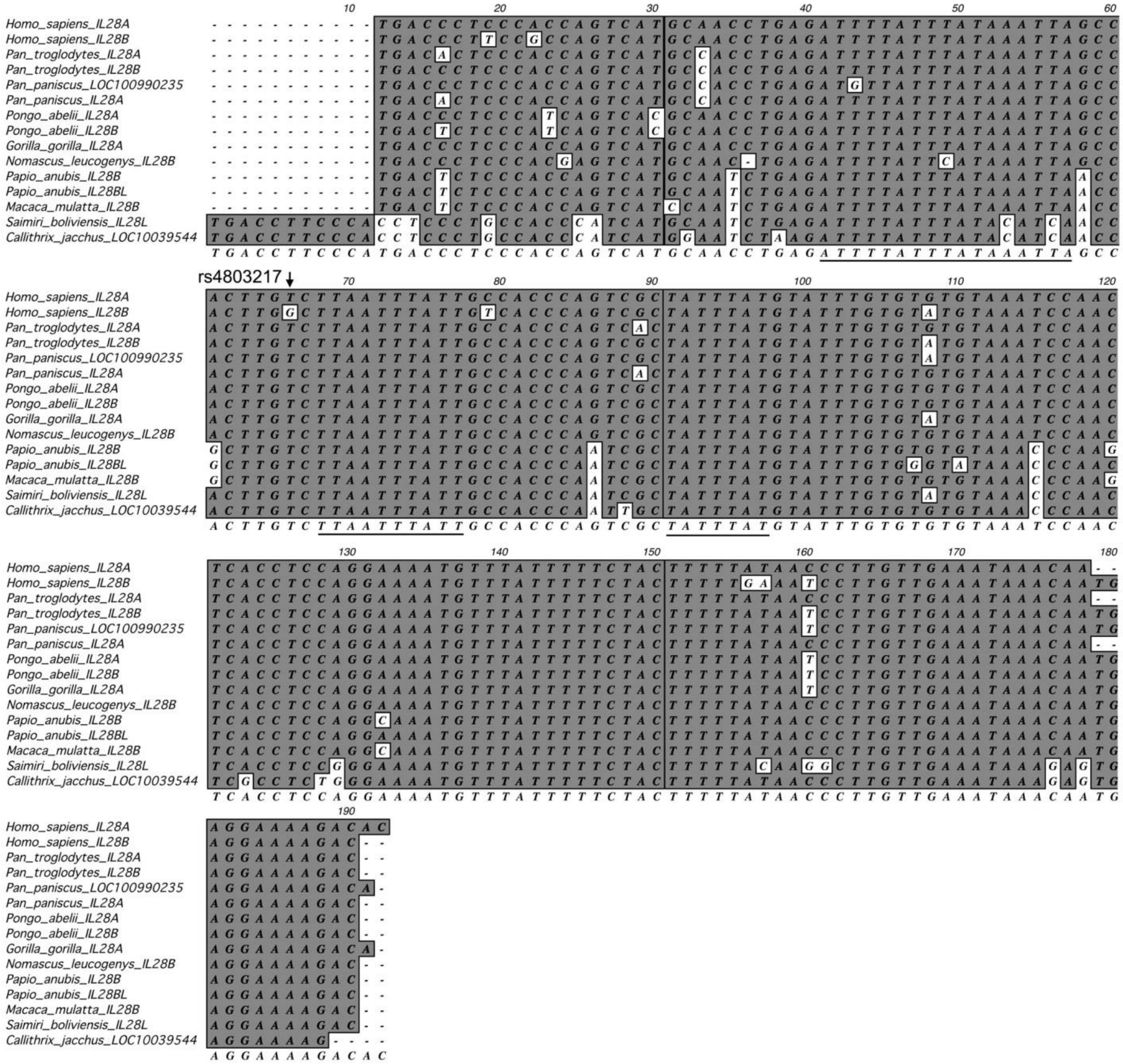

rs4803217
Homo_sapiens_IL28A
Homo_sapiens_IL28B
Pan_troglodytes_IL28A
Pan_troglodytes_IL28B
Pan_paniscus_LOC100990235
Pan_paniscus_IL28A
Pongo_abelii_IL28A
Pongo_abelii_IL28B
Gorilla_gorilla_IL28A
Nomascus_leucogenys_IL28B
Papio_anubis_IL28B
Papio_anubis_IL28BL
Macaca_mulatta_IL28B
Saimiri_boliviensis_IL28L
Callithrix_jacchus_LOC10039544

Figure S2

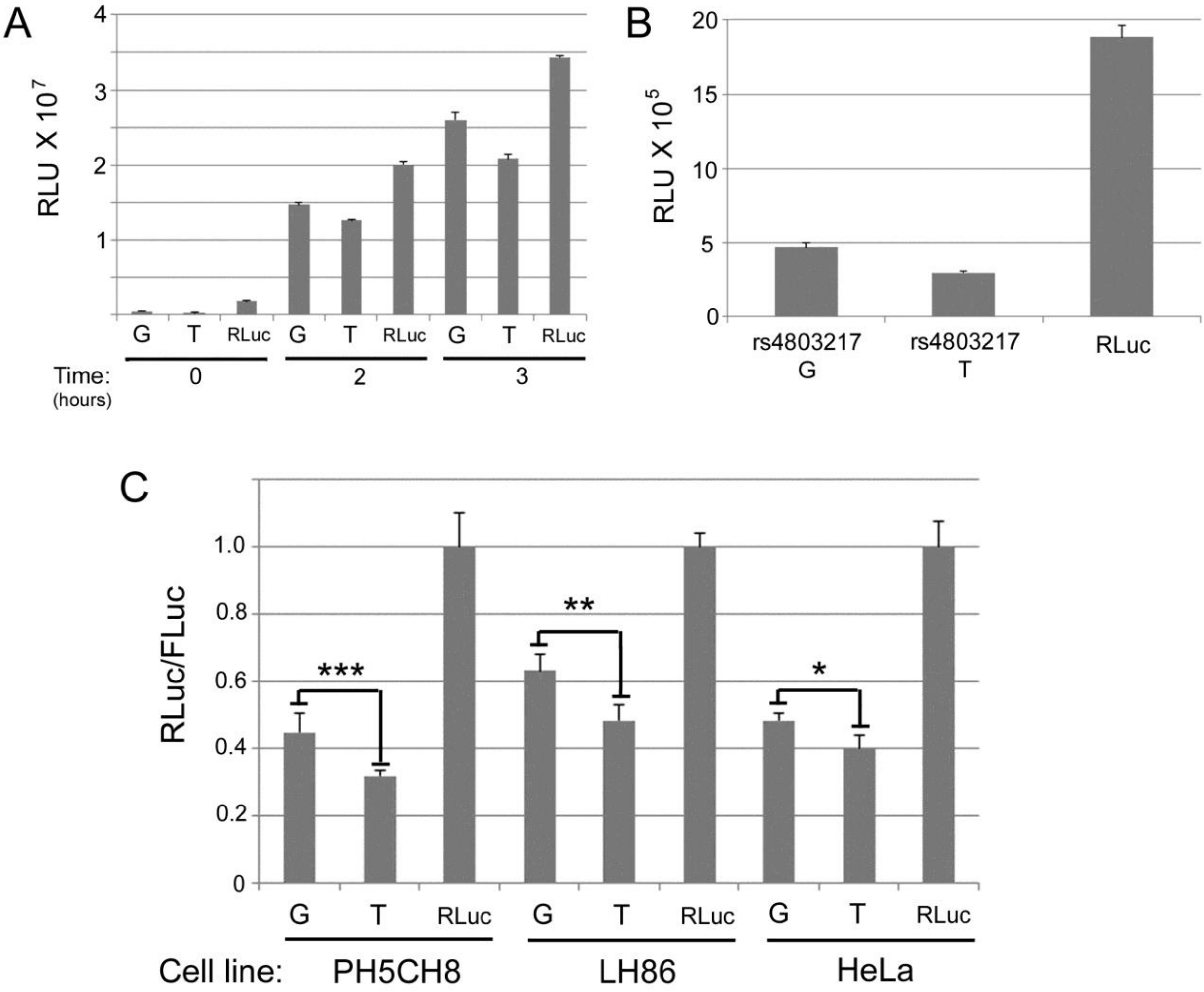
A
RLU X 10^7
4
3
2
1
G
T
RLuc
G
T
RLuc
G
T
RLuc
Time:
(hours)
0
2
3
B
RLU X 10^5
20
15
10
5
0
rs4803217
G
rs4803217
T
RLuc
C
RLuc/FLuc
1.0
0.8
0.6
0.4
0.2
0
***
**
*
G
T
RLuc
G
T
RLuc
G
T
RLuc
Cell line:
PH5CH8
LH86
HeLa

Figure S3

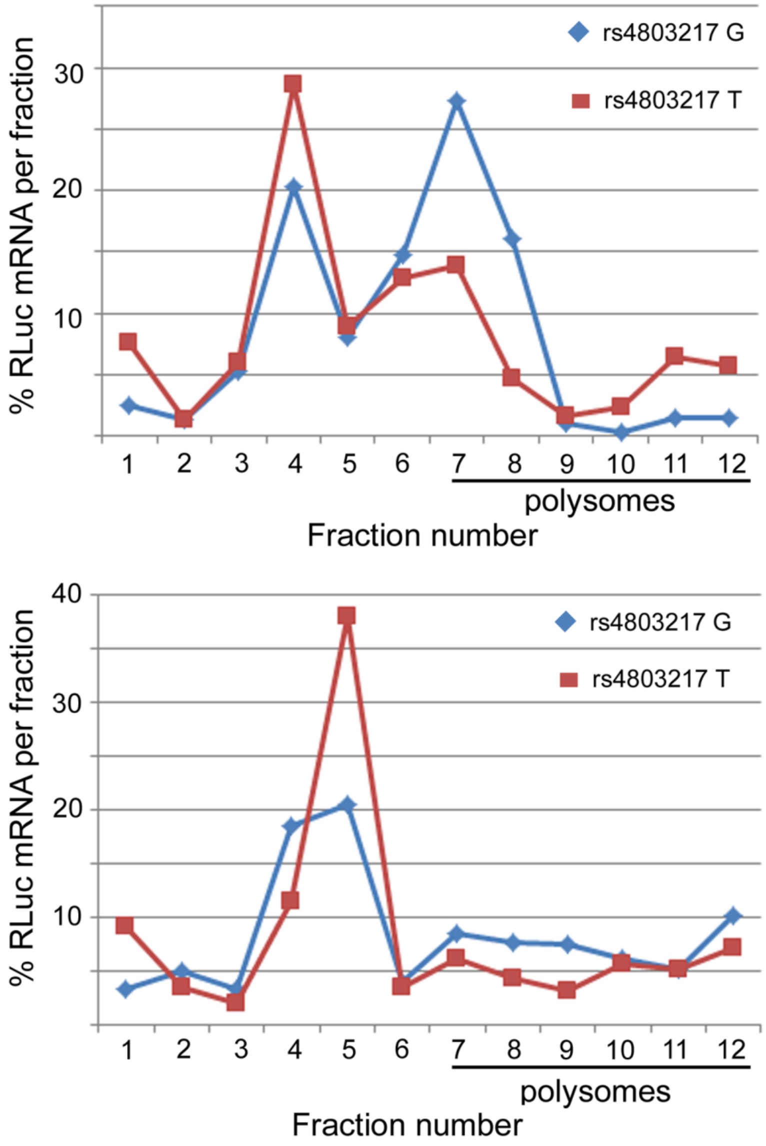
% RLuc mRNA per fraction
30
20
10
rs4803217 G
rs4803217 T
1
2
3
4
5
6
7
8
9
10
11
12
polysomes
Fraction number
% RLuc mRNA per fraction
40
30
20
10
rs4803217 G
rs4803217 T
1
2
3
4
5
6
7
8
9
10
11
12
polysomes
Fraction number

Figure S4

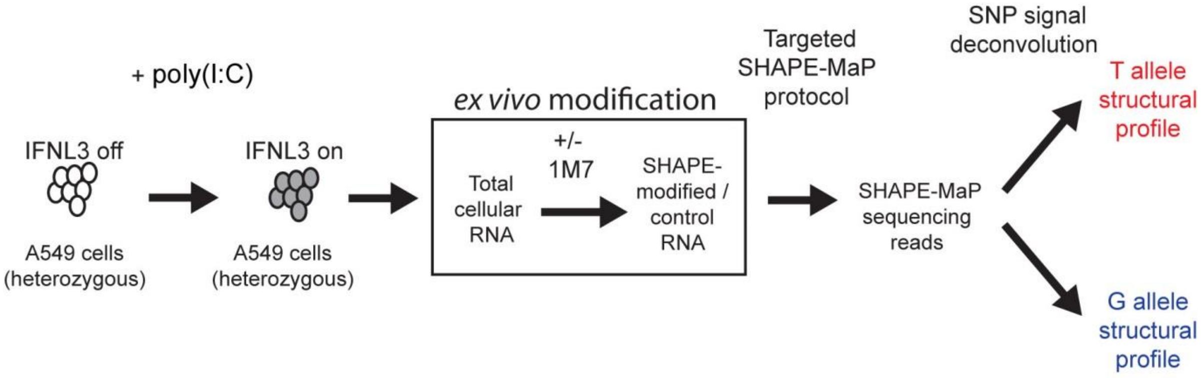
+ poly(I:C)
IFNL3 off
A549 cells (heterozygous)
IFNL3 on
A549 cells (heterozygous)
ex vivo modification
Total cellular RNA
+/- 1M7
SHAPE-modified / control RNA
Targeted SHAPE-MaP protocol
SHAPE-MaP sequencing reads
SNP signal deconvolution
T allele structural profile
G allele structural profile

Figure S5

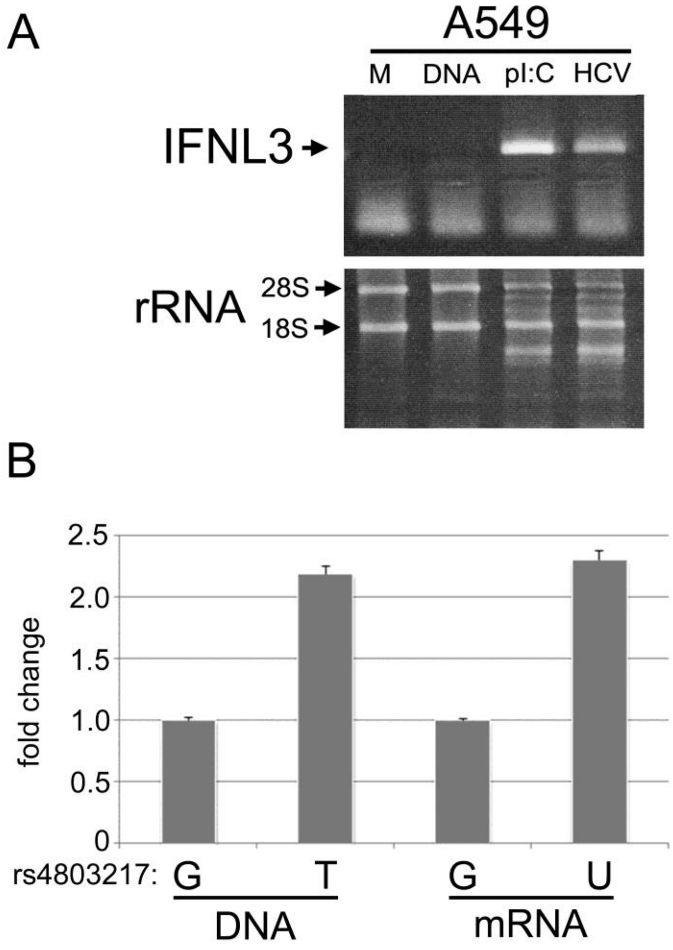
A
A549
M
DNA
pI:C
HCV
IFNL3
rRNA
28S
18S
B
fold change
2.5
2.0
1.5
1.0
0.5
0
rs4803217:
G
T
G
U
DNA
mRNA

Figure S6

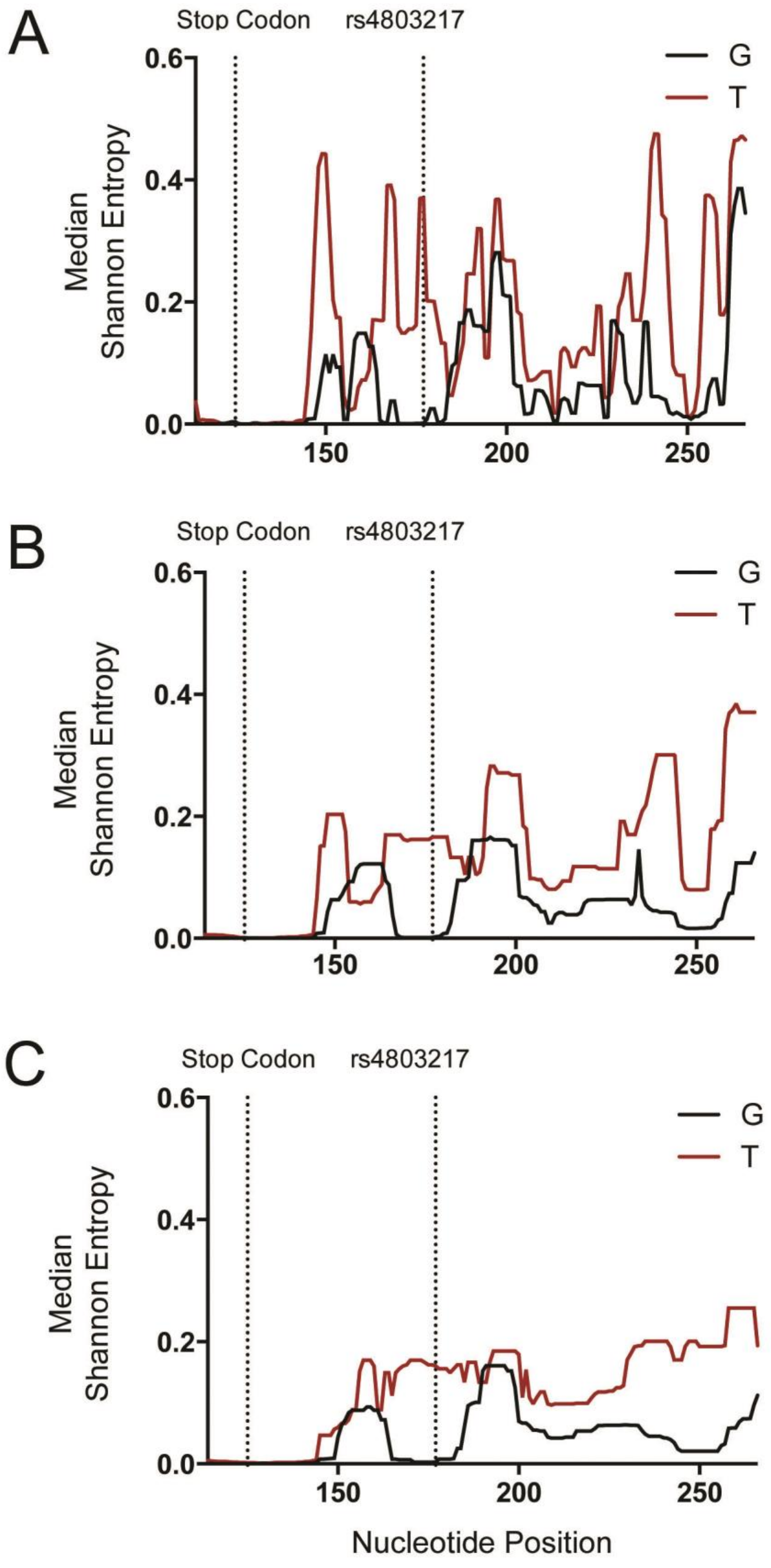
A
Stop Codon
rs4803217
Median Shannon Entropy
0.6
0.4
0.2
0.0
150
200
250
G
T
B
Stop Codon
rs4803217
Median Shannon Entropy
0.6
0.4
0.2
0.0
150
200
250
G
T
C
Stop Codon
rs4803217
Median Shannon Entropy
0.6
0.4
0.2
0.0
150
200
250
G
T
Nucleotide Position

Figure S7

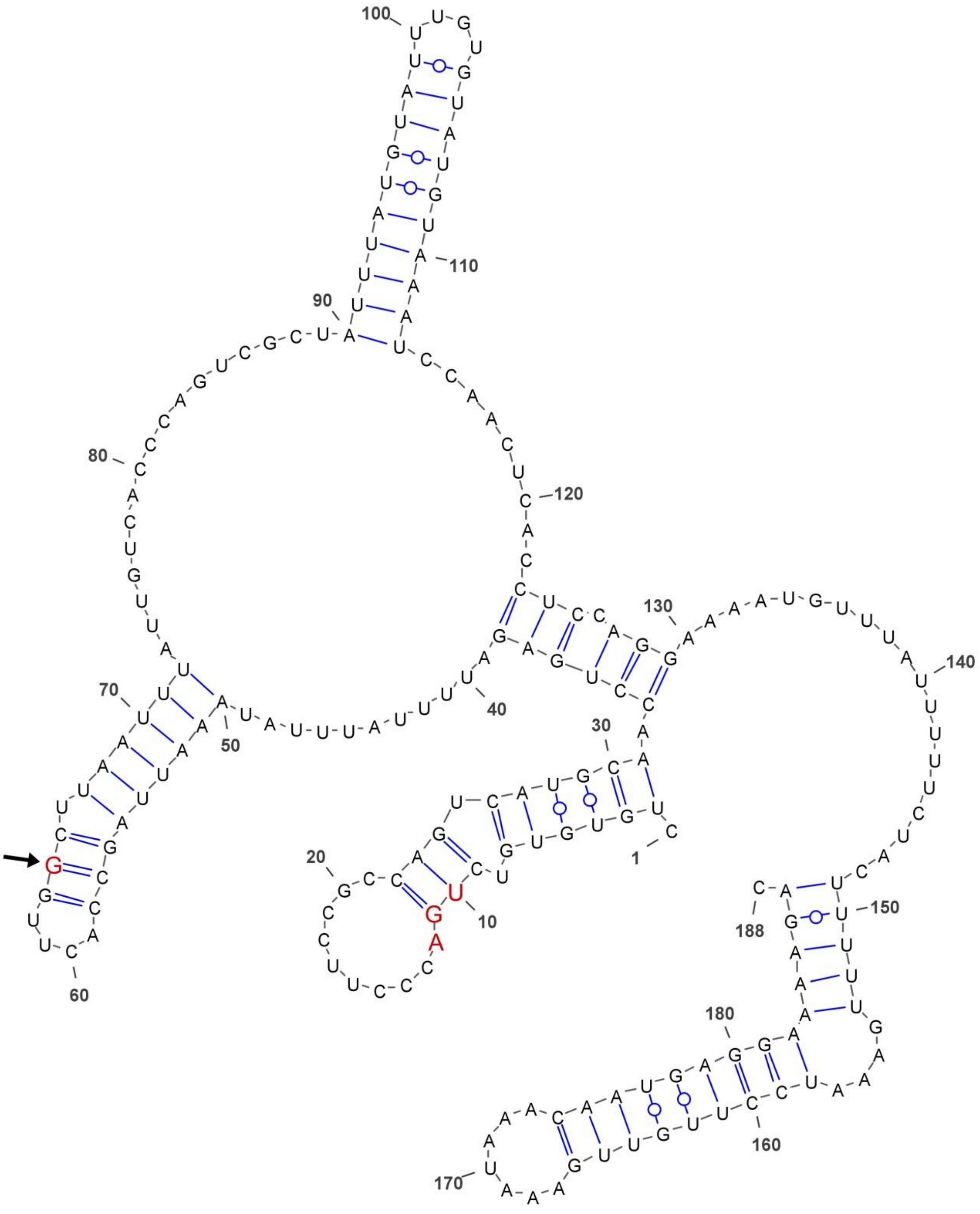

Figure S8

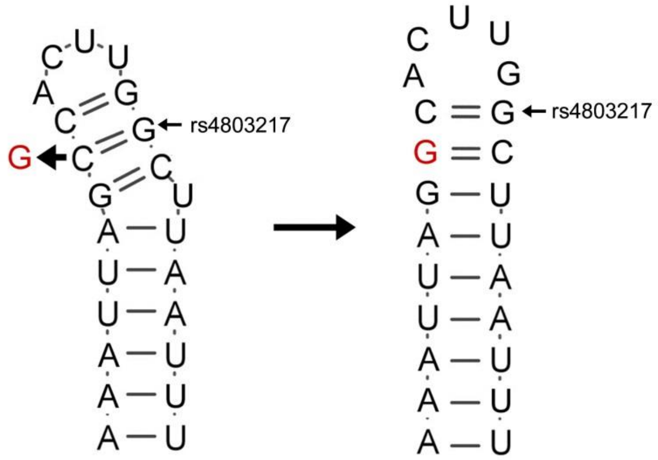

Supplement: Supplementary Information [file srep16037-s1.pdf]
