# Supplementary material for: IFNL3 mRNA structure is remodeled by a functional non-coding polymorphism associated with hepatitis C virus clearance
Source: Sci Rep. 2015 Nov 4;5:16037. doi: 10.1038/srep16037 (PMC4631997; doi:10.1038/srep16037)
Supplement: Supplementary Information [file srep16037-s2.pdf]

Raw SHAPE reactivities

	sequence	rs4803217 G	rs4803217 T
1	A	-0.058307	0.044643
2	C	0.163034	-0.095498
3	C	-0.437721	-0.260724
4	G	0	0
5	G	-0.25759	-0.05544
6	C	0.03409	0.62532
7	T	0	0
8	C	-0.027717	0.723686
9	C	0.227478	-0.162552
10	A	0	0
11	G	0.227941	0.305351
12	G	-0.013796	-0.491233
13	A	-0.045593	0.060919
14	G	-0.348938	-0.711182
15	G	-0.791468	-1.002216
16	C	-0.47306	-0.009033
17	C	-0.117711	-0.086514
18	C	0.574091	-0.055423
19	C	-0.681479	-0.224353
20	A	0.233622	0.266306
21	A	0.55811	0.573367
22	A	0.241081	0.276176
23	A	0.399277	0.889275
24	A	1.278185	0.977057
25	A	0.057207	0.302984
26	G	0.537017	0.217303
27	G	-0.164908	-0.03732
28	A	-0.045436	-0.425863
29	G	-0.495124	-0.027174
30	T	0	0
31	C	0.492105	0.288204
32	C	-0.516846	0.633747
33	C	-0.224789	1.289507
34	C	1.735802	0.207263
35	T	0.027599	0.205257
36	G	0.169028	0.039469
37	G	-0.009125	-0.285275
38	C	0.217265	0.604227
39	T	-0.232587	0.440143
40	G	-0.083709	-0.163417
41	C	-0.123846	0.105399
42	C	0.721896	-0.068558
43	T	0.098088	0.105542
44	C	1.579105	0.87034
45	G	0.037465	0.138956

46	A	-0.185415	0.357104
47	G	-0.325419	0.248293
48	G	-0.234154	-0.173707
49	C	-0.197204	0.439804
50	C	-0.364673	-0.481471
51	T	0.084795	-0.123655
52	C	0.25889	0.875335
53	T	-0.120058	0.72044
54	G	0.661341	0.49774
55	T	0.008587	0.735957
56	C	0.09007	-0.113186
57	A	0.134086	0.040903
58	C	0.523982	-0.156771
59	C	-0.288988	-0.246427
60	T	0.246879	0.691295
61	T	0.635031	0.168582
62	C	0.11259	-0.239836
63	A	0.065313	0.614851
64	A	0.361274	0.865388
65	C	-0.775653	-0.274502
66	C	-0.314996	0.395236
67	T	0.26402	0.144646
68	C	-0.085726	-0.647472
69	T	0.160353	-0.11671
70	T	0.16093	0.756263
71	C	0.204587	0.551633
72	C	-0.385568	-0.280799
73	G	0	0
74	C	0.314854	0.370345
75	C	-0.01221	0.886268
76	T	-0.053276	1.379624
77	C	1.129483	0.521682
78	C	0.080734	1.292831
79	T	1.244031	1.13022
80	C	0.792627	1.211164
81	A	0.323616	0.889185
82	C	0.402042	0.763923
83	G	-0.065853	0.653146
84	C	2.19459	1.222729
85	G	1.165718	0.617749
86	A	0.723979	0.320732
87	G	-0.08413	0.13272
88	A	0.120747	0.258099
89	C	-0.402363	0.364026
90	C	0.590223	1.697097
91	T	0.594884	2.015163
92	G	0.505771	0.941986

93	A	0.97321	0.907594
94	A	2.167485	1.236452
95	T	1.096071	0.639564
96	T	0.233225	0.246273
97	G	0.658279	0.291846
98	T	0.058646	0.591072
99	G	1.077651	0.826507
100	T	-0.430311	0.130412
101	T	0.723663	0.895477
102	G	0.336148	0.765403
103	C	0.913263	0.731931
104	C	0.638247	0.617135
105	A	-0.058516	0.308146
106	G	1.201145	0.772804
107	C	1.205579	0.617125
108	G	0.123138	-0.219705
109	G	0.653839	0.826412
110	G	-0.576624	-0.644003
111	G	0.518139	1.241578
112	A	-0.049632	-0.103574
113	C	0.899877	-0.063279
114	C	0.509315	0.45975
115	T	-1.306303	-0.428717
116	G	0.466457	1.167495
117	T	-0.143998	-0.342636
118	G	0.022914	0.431782
119	T	0.50768	-0.015114
120	G	0.607926	0.812313
121	T	-0.457025	0.47229
122	C	0.323908	0.416621
123	T	0.105211	0.37497
124	G	0.391607	-0.311805
125	A	1.405144	1.057701
126	C	0.343232	-0.60971
127	C	-0.40581	-0.254628
128	C	-0.367625	0.205851
129	T	0.866595	0.552864
130	T	0.096668	1.298862
131	C	0.017585	-0.304657
132	C	1.320541	0.319064
133	G	-0.186732	-0.033646
134	C	1.530169	2.923263
135	C	0.085022	0.499613
136	A	0.611433	-0.111885
137	G	1.056995	-0.238411
138	T	-0.570998	0.636314
139	C	0.090579	0.449312

140	A	0.404559	0.785051
141	T	0.227298	0.179427
142	G	0.434925	0.632644
143	C	0.633794	0.696816
144	A	-0.203285	0.160966
145	A	0.939326	0.256513
146	C	-0.202799	0.955804
147	C	-0.078414	0.621681
148	T	-0.043022	0.352566
149	G	0.248886	0.247861
150	A	-0.849252	0.581837
151	G	-0.003818	0.101284
152	A	0.940812	1.856391
153	T	0.048512	0.053288
154	T	-0.427013	-0.043521
155	T	4.309176	0.919874
156	T	0.209884	0.714578
157	A	2.41558	1.674391
158	T	-0.673106	1.034687
159	T	-0.706914	1.533292
160	T	-0.777643	-0.016482
161	A	1.062438	1.375678
162	T	3.481367	0.465363
163	A	-0.016174	0.245423
164	A	1.140794	1.894566
165	A	0.924096	0.789511
166	T	0.24417	0.482119
167	T	0.319236	0.20629
168	A	-0.221198	-0.285444
169	G	1.202764	0.733176
170	C	0.376383	0.320933
171	C	0.311824	1.060597
172	A	0.43342	0.255369
173	C	1.720269	0.273119
174	T	1.141573	1.191642
175	T	0.846244	0.974621
176	G	-0.160874	-0.402926
177	G	0	0
178	C	0.341749	0.581558
179	T	0.698115	0.009804
180	T	0.142039	2.09628
181	A	0.253191	1.024176
182	A	-0.049265	0.5567
183	T	-0.027626	0.257017
184	T	0.678952	0.22355
185	T	-0.008485	0.983171
186	A	1.20973	1.301559

187	T	1.204483	0.370438
188	T	0.626027	0.37268
189	G	0.591424	0.43117
190	T	-0.167665	0.199125
191	C	0.802219	0.601481
192	A	0.385316	0.397238
193	C	-0.253374	-0.136338
194	C	0.210269	0.614671
195	C	0.005922	1.587471
196	A	0.463785	0.451964
197	G	0.250875	0.397766
198	T	1.162025	0.121737
199	C	0.375354	0.418628
200	G	0.242815	0.442185
201	C	1.052008	1.823644
202	T	0.124868	0.569742
203	A	0.064325	0.143075
204	T	-0.271914	0.063677
205	T	0.32649	0.994521
206	T	0.191759	0.753137
207	A	1.441154	0.484805
208	T	1.911027	1.184443
209	G	1.029237	1.083196
210	T	-0.133517	-0.606856
211	A	0.552786	1.296013
212	T	-0.92074	1.161395
213	T	10.215613	7.801866
214	T	0.324667	0.332855
215	G	0.774434	0.551173
216	T	0.579099	0.411962
217	G	-0.009986	0.428961
218	T	-0.143875	0.391578
219	A	0.617273	0.1751
220	T	-0.773869	-0.02665
221	G	0.199213	0.333854
222	T	-0.63952	-0.820321
223	A	-0.162889	-0.089279
224	A	0.515765	0.774299
225	A	0.111481	0.395721
226	T	0.076128	-0.001245
227	C	0.524662	0.832259
228	C	0.511434	1.088499
229	A	-0.005372	0.881642
230	A	0.028911	0.906292
231	C	1.062598	0.677684
232	T	0.034959	0.455633
233	C	0.510957	0.690842

234	A	-0.035324	-0.094059
235	C	0.347261	0.1771
236	C	0.357585	0.377146
237	T	-0.14626	-0.070968
238	C	1.709499	1.664404
239	C	1.058896	0.22641
240	A	0.173264	0.074313
241	G	-0.018687	-0.027344
242	G	0.149108	-0.053312
243	A	0.085159	0.16582
244	A	2.331194	1.0395
245	A	1.021608	0.906319
246	A	1.956118	1.609939
247	T	0.051614	0.817769
248	G	0.595422	0.575161
249	T	1.482186	-0.246782
250	T	1.419318	1.74219
251	T	1.965305	2.276584
252	A	1.452571	1.15907
253	T	0.564469	0.426281
254	T	-0.38125	0.564067
255	T	1.274329	-0.192518
256	T	1.120275	0.031819
257	T	0.51627	0.186158
258	C	0.38249	0.702996
259	T	0.119497	0.016727
260	A	0.535979	1.359437
261	C	0.760772	0.163294
262	T	-0.238715	0.181353
263	T	0.366675	0.260721
264	T	-0.417367	-0.121565
265	T	-0.74785	-0.214342
266	T	-0.1033	-0.04916
